# Application of flexible fiberoptic bronchoscopy in the removal of adult airway foreign bodies

**DOI:** 10.1186/s12893-020-00825-5

**Published:** 2020-07-23

**Authors:** Weijun Ma, Juan Hu, Miaoli Yang, Yeye Yang, Min Xu

**Affiliations:** Department of Otolaryngology Head and Neck Surgery, Second Affiliated Hospital of Xi’an Jiaotong University, No. 157 Xiwu Road, Xi’an, 710004 Shaanxi China

**Keywords:** Airway foreign body, Adult, Flexible fiberoptic bronchoscope

## Abstract

**Background:**

Flexible fiberoptic bronchoscopy is a rapid, cost effective and safe procedure.

**Aim:**

To analyze demographic information and endoscopic findings in adult patients with airway foreign body aspiration and its removal.

**Methods:**

Fifty-seven adults (40 males, 17 females; average age 40 years old) with airway foreign body aspiration were analyzed. Cough (37, 65%) was the most common clinical presentation. The most common foreign body was bone followed by dental prosthesis and food debris.

**Results:**

In the current study, 42 out of the 57 (74%) airway foreign bodies were successfully removed under flexible fiberoptic bronchoscopy. However, it was failed in 15 patients and thus, rigid bronchoscopy was used to remove foreign bodies successfully in 13 of the 15 patients. Thoracotomy was performed for the 2 patients whose foreign body removal was unsuccessful even with rigid bronchoscopy.

**Conclusion:**

The findings of the current study revealed that flexible fiberoptic bronchoscopy is a safe and effective procedure for the removal of adult airway foreign bodies in the majority of cases. Rigid bronchoscopy can be a backup procedure in case flexible bronchoscopy is failed.

## Background

Aspiration of a foreign body into the tracheobronchial tree is a common event in children, especially in those younger than 3 years of age [1]. Although it is less common, aspiration of a foreign body in adults may also occur. Risk factors such as neurodegenerative or neuromuscular disease may contribute to foreign body aspiration in adults [2]. Chronic cough and hemoptysis may be present in adult patients with airway foreign body aspiration, but many patients are asymptomatic. Therefore, aspiration of a foreign body in adult is frequently misdiagnosed. Delay in diagnosis and treatment of airway foreign body aspiration may result in complications such as pneumonia and atelectasis [3–5].

Foreign body aspiration in adult may be managed by either rigid or flexible fiberoptic bronchoscopy, depending on the location of the foreign body and practice patterns. While rigid bronchoscopy has been used to remove the airway foreign body in adults, there is no gold standard of the procedure. Flexible fiberoptic bronchoscopy is a rapid, cost effective and safe procedure. Therefore, in the current study, flexible fibrotic bronchoscopy was chosen as first procedure to remove the airway foreign body in adult and rigid bronchoscopy was used as a second line procedure. Here, we summarized the clinical manifestations, diagnosis, and removal of adult airway foreign bodies that were treated in our hospital from 2002 to 2015.

## Methods

Medical history records of the patients with airway foreign body aspiration, who visited Department of Otolaryngology-Head & Neck Surgery at The Second Affiliated Hospital of Xi’an Jiaotong University, Shaanxi, China from 2002 through 2015, were retrospectively reviewed. Study protocol was approved by The Institutional Review Board of The Second Affiliated Hospital of Xi’an Jiaotong University.

Fifty-seven cases of airway foreign body removal were reviewed. Flexible fiberoptic bronchoscopy was applied as the first line procedure in all 57 cases. To perform the bronchoscopy, atropine (0.5 mg) and diazepam (10 mg) were given as premedication 30 min before the procedure. Topical anesthesia with 2% lidocaine and 1% ephedrine was delivered through the nose, followed by bilateral superior laryngeal nerve block and endotracheal anesthesia with 2% lidocaine. The procedure was performed at supine position, and a flexible fiberoptic bronchoscope, foreign body forceps, biopsy forceps (Olympus, Tokyo, Japan), and an aspirator were used. A rigid bronchoscope was available as a backup device. The procedure was performed under mild sedation and spontaneous ventilation for the patient. Occasional bleeding, when present, was controlled with topical instillation of epinephrine.

**Fig. 1:**
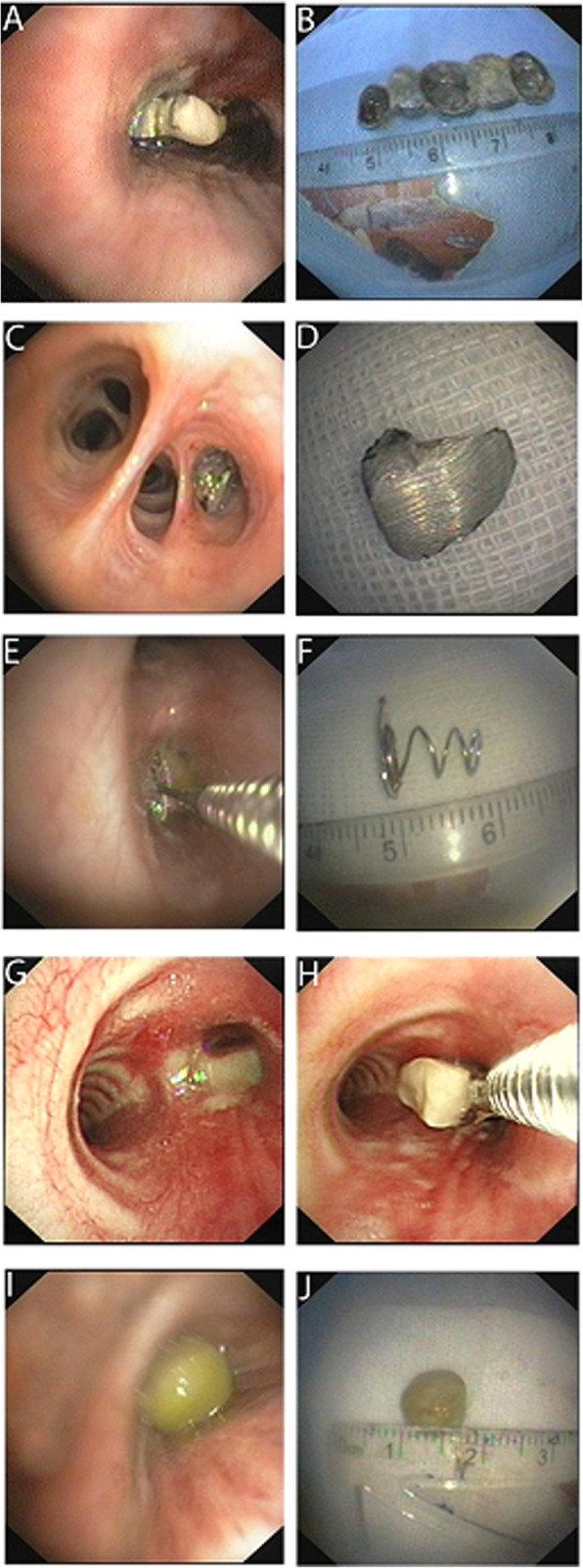
Images of aspirated foreign bodies. **a & b**: Bronchoscopy view of a dental prosthesis in the right middle lobe bronchus. **c & d**: Bronchoscopy view of part of a dental prosthesis in the right lower lobe bronchus. **e & f:** Endoscopic image taken during the removal of a metal spring from the right bronchus. **g & h**: Endoscopic image taken during the removal of a peanut from the left lower lobe bronchus. **i & j**: Bronchoscopy view of a whole soybean in the right lower lobe bronchus

Patients’ general information including age, gender, symptoms, foreign body characteristics, foreign body location, and complications were reviewed. Of the 57 patients, 40 were male (70.1%) and 17 were female (29.8%). Their ages ranged from 14 to 73 years old (mean, 39.7 years old). The most common symptoms were cough (37, 64.9%) followed by hemoptysis (23, 40.3%), wheezing (20, 35.1%), fever (16, 28.1%), vomiting (13, 22.8%), and dyspnea (6, 10.5%). The most common aspirated foreign bodies were bone (18, 31.6%, Table 2) followed by dental prosthesis (14, 24.6%; Fig. 1a-d and Table 2), metallic objects (6, 10.5%; Fig. 1e & f and Table 2), food and nut (14, 24.6%; Fig. 1g-j and Table 2), medication tablet (3, 5.3%, Table 2), pen cap (1, 1.7%, Table 2), and pin (1, 1.7%, Table 2). The foreign body was located in the right bronchus in 45 patients, and in the left bronchus in 12 patients (21.1%).

The most common findings on the chest radiograph were pulmonary and pleuropulmonary infiltration (46.1%). Other findings were atelectasis in the segmental or lobar bronchi (10, 17.2%), space occupying lesion (2, 3.5%), and lung abscess (2, 3.5%). Normal chest radiograph was shown in 14 patients (25.1%). Diagnosis of foreign body aspiration was confirmed, and the foreign body was immediately removed under flexible fiberoptic bronchoscopy within 24 h in 8 cases. In the remaining 49 cases, foreign body aspiration was diagnosed within 2 to 30 days after the event in 43 cases, and 1 to 12 months or longer after the event in 6 cases. The longest time between aspiration and removal was 3 years, which was a case of 51-year-old man who did not see a doctor during the 3 years. Two patients could not recall when the foreign body was aspirated into the airway.

While the majority of the patients enrolled in this study (39, 68.4%) had no known risk factors or comorbidities, as shown Table 1, the following comorbidities were identified and they could be the risk factors for the foreign body aspiration: neurodegenerative diseases (7, 12.3%), cerebrovascular disease (5, 8.7%), drug addiction (4, 7.0%), and mental illness (2, 3.5%).

**Table 1:** Comorbidities of the patients

Comorbidity	Number (%)
Neurodegenerative disease	7 (12.3)
Cerebrovascular disease	5 (8.7)
Drug addiction	4 (7.0)
Mental illness	2 (3.5)

The majority of the patients had no complications. Sixteen of the 57 patients (28.1%) were complicated with pneumonia and only 2 (3.5%) had atelectasis. However, granulation tissue and mucosal inflammation were found in most of the patients and only 3 had normal mucosa. Complications of foreign body aspiration were associated with type of the foreign body and the interval between aspiration and removal. Vegetable or organic materials could cause a higher rate of complications in that proteins or lipids could lead to more severe inflammation and tissue injury. The longer the foreign body stayed inside the airway, the higher complication rate was.

**Fig. 2:**
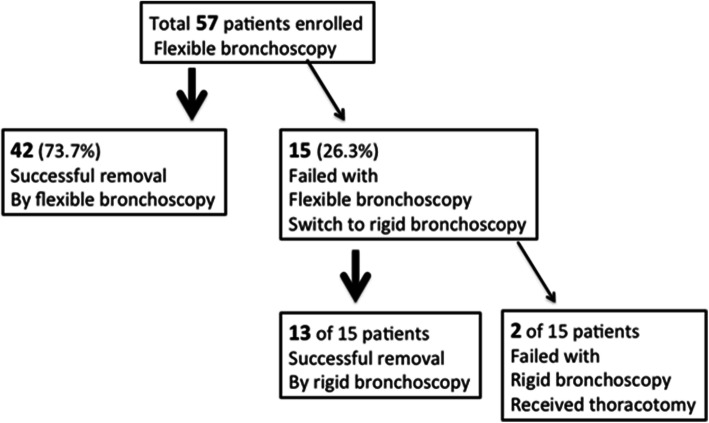
Diagrammatic outline of the number of patients treated with flexible or rigid bronchoscopy and thoracotomy

As outlined in Fig. 2, flexible fiberoptic bronchoscopy was first applied in all of the patients because they were stable without asphyxia. The foreign body was successfully removed under flexible fiberoptic bronchoscopy in 42 of the 57 patients (73.7%). In 15 out of the 57 patients, however, removal of the foreign bodies was failed with flexible fiberoptic bronchoscope, and thus, a rigid bronchoscopy was performed, by which, foreign bodies were successfully removed in 13 out the 15 patients. The remaining two patients, in whom neither flexible nor rigid bronchoscopy was successful, underwent thoracotomy for removal of the foreign bodies. Specifically, as shown in Table 2, overall successful rate of foreign body removal with flexible bronchoscopy was 73.7%. Majority of the dental prosthesis (10 out of 14 cases) were unsuccessfully removed with flexible fiberoptic bronchoscopy (only 3 out of 14 were successful), while all of the bone (18 out of 18) or food and nuts (14 out of 14) were successfully removed with flexible fiberoptic bronchoscopy.

**Table 2:** Successfully removed foreign bodies and case number by different methods

Foreign bodies	Flexible	Rigid	Thoracotomy
Bone	18		
Dental prosthesis	3	10	1
Metallic objects	4	2	
Food or nuts	14		
Medication tablet	3		
Pen cap			1
Pin	1		

## Discussion

The present study retrospectively summarized risk factors, features, and management of foreign body aspiration in 57 adult patients. The age and gender characteristics of the patients enrolled in this study were similar to that of previously reported studies [4, 6].

Patients with neurologic and neuromuscular disease, head trauma or alcohol addiction were more susceptible to foreign-body aspiration [3, 7]. Consistently, neurodegenerative disease, cerebrovascular disease, drug addiction, and mental illness were predominant risk factors in one-third of the cases in the current study, although no definite associations with such risk factors were identified in 39 (68.4%) of the 57 patients.

In the current study, we found that drug addiction was the main risk factor for foreign body aspiration in patients under 15 years old. In contrast, cerebrovascular disease was the major cause in patients older than 70 years, although ill-fitting dentures and inattentiveness during eating or drinking might also contribute to foreign body aspiration in elder patients. These findings indicated that drug addiction in youngers and neuromuscular disease in elders could be risk factors of airway foreign body aspiration.

While the most common symptoms in children with foreign body aspiration were choking, breathlessness, cough and wheeze [8], adults were often asymptomatic or had mild or non-specific symptoms such as chronic cough, dyspnea on exertion, and hemoptysis [8]. In adults with foreign body aspiration, the lack of a precise history and paucity of symptoms often resulted in misdiagnosis or delayed diagnosis [9–11]. Consistently, most of the cases in the current study had mild symptoms including cough, hemoptysis, wheezing, vomiting, fever and dyspnea.

The most frequently identified foreign body in this study was bone followed by food (including nut), dental prosthesis, and metallic objects, which was similar to the earlier reports by others [12]. Interestingly, however, the types of foreign bodies varied in different age groups. In the older patients, dental prosthesis and food debris were commonly identified, whereas pen caps and metallic objects were more common in younger age group.

Although only chest radiograph was used for diagnosis in the present study, a computed tomography scan is recommended to visualize the airway foreign body location and size, as it is more sensitive and specific for the diagnosis of radiolucent foreign bodies and for determining the characteristics of a suspected foreign body [8, 13–15].

The bronchoscope was introduced in 1897 by the otolaryngologist Gustav Killian for the removal of a foreign body in the airway. He used a rigid bronchoscope for the purpose [16]. Shigeto Ikeda developed the flexible bronchoscope in 1968. Zavala and Rhodes [17] performed animal studies to show that a flexible fiberoptic bronchoscope could be used to remove various types of foreign bodies using forceps. Since then, the flexible fiberoptic bronchoscope has been increasingly used for the diagnosis and removal of foreign bodies in stable patients without asphyxia, using combinations of forceps, snares, or baskets. In contrast to the rigid bronchoscope, flexible fiberoptic bronchoscopy is easier, less expensive, and does not require a general anesthesia. In addition, preservation of the cough reflex with mild sedation is potentially helpful for the removal of a foreign body that may be dislodged during the procedure.

Successful rate of foreign body removal with flexible fiberoptic bronchoscopy was largely different in the literature. To our knowledge, the highest successful rate was reported by Dong et al. with 96.5% successful in 200 cases [8] followed by Tang et al. with 91.3% success in 1027 children [18] and by Mise et al. with 90.7% success in 86 cases [3]. Rodrigues [19] reported a success rate of 82.5% with the use of a flexible bronchoscope for the removal of foreign bodies in 33 patients. Limper and Prakash [20] had a success rate of 60% with the flexible bronchoscope. The present study showed a success rate of 73.7% with first line application of the flexible fiberoptic bronchoscope. Findings of the current study indicated that the success rate of foreign body removal with flexible fiberoptic bronchoscopy seemed largely associated with the type and shape of the foreign body. In this regard, majority of dental prosthesis removal were failed by flexible fiberoptic bronchoscopy in the current study, and thus, we recommend that rigid bronchoscopy should be used as first choice for dental prosthesis removal. In contrast, all of bone and food debris were successfully removed with flexible fiberoptic bronchoscopy, suggesting flexible fiberoptic bronchoscopy under local anesthesia could be the first choice for this type of foreign bodies. In addition, choice of rigid or flexible bronchoscopy may also depend on comorbidity of respiratory diseases. In this regard, for patients who present with respiratory failure, attempt with flexible bronchoscopy may be hazardous, and rigid bronchoscopy is a quicker and safer procedure in these patients [21]. Thus, rigid bronchoscopy should be the preferred upfront modality for foreign body removal in patients with respiratory failure.

For the removal of a large object, foreign body forceps were used to grasp the object which was then slowly removed together with the bronchoscope. For small foreign bodies such as food debris, biopsy forceps and negative pressure suction were used. When the foreign bodies were beyond the vocal cords, the patient was instructed to take a deep breath and hold it for a moment so that the passage would open more widely. Anesthesia for rigid bronchoscopy and thoracotomy backup were always ready immediately in case of asphyxiation.

## Conclusion

Foreign-body aspiration in adults was relatively rare and the patients were frequently asymptomatic or had mild symptoms. Suspicion of foreign body aspiration is highly recommended for prompt diagnosis in adults with neuromuscular disease or drug addiction. While chest X-ray could be a primary procedure for diagnosing airway foreign body aspiration, computed tomography scan is highly recommended to visualize the exact location and size of the foreign body. Flexible fiberoptic bronchoscopy under local anesthesia can be used as a primary procedure for the removal of the airway foreign body. Rigid bronchoscopy can be an alternative and second choice for the removal of majority airway foreign bodies such as bone and food, but should be the first choice for the removal of dental prosthesis in adult.

## Data Availability

The datasets generated and analyzed during the current study are available from the corresponding author on reasonable request.
